# Genetics and genomic medicine in Argentina

**DOI:** 10.1002/mgg3.571

**Published:** 2019-02-05

**Authors:** Javier Cotignola, Sandra Rozental, Noemí Buzzalino, Liliana Dain

**Affiliations:** ^1^ Laboratorio de Inflamación y Cáncer Departamento de Química Biológica Facultad de Ciencias Exactas y Naturales Universidad de Buenos Aires Buenos Aires Argentina; ^2^ IQUIBICEN CONICET Buenos Aires Argentina; ^3^ Centro Nacional de Genética Médica ANLIS Buenos Aires Argentina; ^4^ Facultad de Ciencias Exactas y Naturales Universidad of Buenos Aires Buenos Aires Argentina

**Keywords:** argentina, diagnosis, genetics, genomics

## Abstract

In this letter, we want to add information to the paper “Genetics and genomic medicine in Argentina” that we considered it was lacking. Argentina is a big country with inequalities in the access to public health care, especially in medical genetics and genomics.

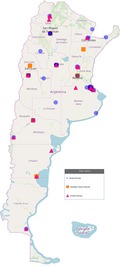

## To the Editor,

The paper “Genetics and genomic medicine in Argentina” by Vishnopolska et al. (2018) is a good initiative to describe the situation of the field in the country. A detailed demographic and economic situation is presented, and some important milestones describing the history and development of some genetic areas are well described. Nevertheless, we consider that the paper lacks important additional information about the current situation of genetic and genomic medicine in Argentina. In this letter, we want to add information of some other public resources to provide the reader with a broader picture.

The authors described the status of genetic services in Argentina based on a census published in 2006 (Liascovich, Rozental, Barbero, Alba, & Ortiz, 2006). However, there is newer data reporting 45 genetic services and 51 laboratories in public Institutions in 18/24 Argentina provinces (http://www.anlis.gov.ar/cenagem/?page_xml:id=119; last accessed August 2018). Altogether, these Services provide biochemical, cytogenetic, and molecular studies (from single‐gene analysis to NGS, FISH, MLPA, and SKY) for at least 76 different genetic disorders. In addition, the Ministry of Health created the Program for Rare Diseases and Congenital Abnormalities in 2014 under the National Law 26.689/2011 (http://www.msal.gov.ar/congenitas). This Program aims to raise awareness and training in rare genetic diseases and congenital birth defects. It also compiles information about public resources in the country. There are also 20 public forensic genetic services (www.mincyt.gob.ar/_post/descargar.php?idAdjuntoArchivo=35074; last accessed: August 2018).

We agree with Vishnopolska et al. that these days more laboratories are using NGS for genomic diagnosis. But we believe that it is equally important to mention that array‐CGH was first incorporated in 2014 at CENAGEM for the study of CNVs at a genomic scale. (http://www.anlis.gov.ar/cenagem/wp-content/uploads/2017/11/Laboratorios-Argentina-2017-1.pdf). Moreover, other public Institutions are also introducing array‐CGH for studying genomic imbalances in genetic disorders.

The Argentine Hereditary Cancer Network (RACAF) has been in operation since 2013 as part of the National Program of Familial and Hereditary Tumors (PROCAFA) belonging to the National Cancer Institute of the Argentine Ministry of Health. By the end of 2017, the RACAF was comprised by 24 public and 34 private genetic counseling clinics spread in 11 provinces (http://www.msal.gob.ar/images/stories/bes/graficos/0000001236cnt-20180705-reporte-anual-racaf-2017.pdf). In 2017, the SITHER (Hereditary Tumor Information System) was publicly launched by the RACAF as an open‐access source of anonymized genomic and epidemiological information for Hereditary Cancers (SITHER website: https://bit.ly/2wLRHjX). The SITHER is a public repository containing the results of molecular studies (mainly NGS and MLPA) from over 500 argentine patients evaluated by RACAF (last accessed: August 2018).

It is also worth to mention other sources of academic translational research focused on genetics and genomics medicine. Since 2013, the National Agency for the Promotion of Science and Technology (ANPCyT) started to fund Biotechnology Projects for Translational Research (PBIT) (http://www.mincyt.gob.ar/convocatoria/fs-2013-biotecnologia-pbit-9024) and Clinical Projects for Development and Investigation (PIDC: http://www.mincyt.gob.ar/convocatoria/pid-2012-clinicos-7966) to be carried out in public Institutions.

Finally, we would like to remark that several research/clinical groups are using genomic approaches for the study and diagnosis of genetic disorders such as neurological disorders (Cordoba et al., 2018; Rodriguez‐Quiroga et al., 2015), Duchenne/Becker muscular dystrophy (Luce et al., 2018), breast cancer (Solano et al., 2017, 2018), ovarian cancer (Cardoso et al., 2018), and endocrine/immune diseases (Gutierrez et al., 2018), among others.

We believe that these data adds information to the article by Vishnopolska et al. We are also aware that there might be other public Institutions where genetic and genomic diagnosis and research are performed. We excluded all nonprofit and private laboratories on this report because this letter was intended to show only the public genetic and genomic medicine landscape in Argentina supported by Government funds. But it is of our knowledge that there are many nonprofit and private Institutions performing similar types of studies.

As a concluding remark, we agree with Vishnopolska et al. that Argentina is a big country with inequalities in the access to public health care, especially in medical genetics and genomics. We also agree that there are programs that aim to train health‐care professionals in medical and molecular genetics/genomics. But there is still a lot of work ahead to be done to bring genetic counseling and genetic/genomic studies to the entire country.

## CONFLICT OF INTEREST

The authors declare no conflict of interest.
